# Pentazocine Protects SN4741 Cells Against MPP^+^-Induced Cell Damage via Up-Regulation of the Canonical Wnt/β-Catenin Signaling Pathway

**DOI:** 10.3389/fnagi.2017.00196

**Published:** 2017-06-14

**Authors:** Jiancai Wang, Jintao Gu, Hao Wu, Gang Zhu, Dayun Feng, Yuqian Li, Wei Guo, Keyong Tian, Guodong Gao, Li Gao

**Affiliations:** ^1^Department of Neurosurgery, Tangdu Hospital, Fourth Military Medical UniversityXi’an, China; ^2^State Key Laboratory of Cancer Biology, Biotechnology Center, School of Pharmacy, Fourth Military Medical UniversityXi’an, China; ^3^Department of Otolaryngology, Xijing Hospital, Fourth Military Medical UniversityXi’an, China

**Keywords:** β-catenin, Parkinson’s disease, pentazocine, MPP^+^, Dkk1

## Abstract

The Wnt/β-catenin signaling pathway has been linked to many neurodegenerative diseases including Parkinson’s disease (PD). A glycoprotein named Dickkopf-1 (Dkk1) can combine with the receptor complex on cell membrane to inhibit Wnt/β-catenin signaling. Opioids, a series of compounds including morphine, fentanyl and pentazocine, have been reported to contribute to the up-regulation of Wnt/β-catenin signaling. Naloxone is an antagonist that has been used as an antidote to opioids through mu-opioid receptor. 1-methyl-4-phenylpyridinium (MPP^+^), which serves as a selective toxin for dopaminergic neurons, has been used to create experimental models of PD. In our study, we examined the protective effects of pentazocine against MPP^+^-induced cell death in the nigral dopaminergic cell line, SN4741 and tried to elucidate the molecular mechanisms underlying such protective effects. The data showed that pretreatment with pentazocine significantly rescued the SN4741 cell against MPP^+^. Moreover, the MPP^+^-exposed SN4741 cells exhibited a down-regulation of β-catenin, which could be restored by treatment with pentazocine. However, Dkk1 but not naloxonewas associated with the abrogation of protective effect of pentazocine. These results suggest that pentazocine alleviates MPP^+^-induced SN4741 cells apoptosis via the up-regulation of canonical Wnt/β-catenin signaling.

## Introduction

Parkinson’s disease (PD) is a common neurodegenerative disorder that is characterized by selective death of dopamine neurons in the substantia nigra (Fearnley and Lees, 1991). However, the molecular mechanism underlying the degeneration of dopamine neurons remains unknown. SN4741 is a nigral dopaminergic cell line. Treating SN4741 cells with 1-methyl-4-phenylpyridinium (MPP^+^), a selective toxin for dopamine neurons, has been used to establish the *in vitro* model of PD (Cai et al., 2015). Recent studies have revealed that downregulation of the Wnt/β-catenin signaling is involved in the development of PD. For example, the down-regulation of p-Ser9-GSK3β has been reported in 6-OHDA-induced parkinsonism models (Chen et al., 2004; Duka et al., 2009). Change of β-catenin in the ventral midbrain of MPTP-induced animal PD models also has been reported (L’Episcopo et al., 2011).

Wnt signaling regulates many important facets of brain development, including the cell proliferation and tissue homeostasis in the central nervous system. There are three different Wnt signaling and the canonical Wnt signaling is the most important one. In the canonical Wnt signaling, Wnt binds to frizzled (FZD) receptor and to LDL receptor-related protein 5/6 (LRP5/6; L’Episcopo et al., 2011; Zhou et al., 2016; Wang et al., 2017), thus activating a series of downstream cascades including the inhibition of GSK-3β by phosphorylation of ser9 and the accumulation of β-catenin (Fukumoto et al., 2001). Then the up-regulation of β-catenin leads to the formation of TCF/LEF-β-catenin complex and the expression of downstream genes including c-Myc which acts as a cell cycle related gene and cyclinD1 which is discovered in embryos and tumor tissues (Udhayakumar et al., 2007; Kim et al., 2010; Li et al., 2013). Dickkopf-1 (Dkk1), a glycoprotein with two cysteine-rich domains, combines with LRP6 of the receptor complex to inhibit Wnt/β-catenin signaling (Cao et al., 2015).

Pentazocine, a weak agonist of the μ-type opioid receptor and strong agonist of the κ- and δ-receptors, is used for the management of a series of pain in animals and humans (Robinson et al., 2016). As a weak agonist, pentazocine shows less chance of addiction compared to morphine and fentanyl that show robust addiction in response to the agonist treatment. Previous studies have indicated that the Wnt/β-catenin signaling can be activated by treatment with opioids (Dunbar et al., 2006). Besides, the opioids have many beneficial roles against damage inside the central nervous system. Elyasi et al. (2014) reported that morphine can protect the SH-SY5Y human neuroblastoma cells against 6-OHDA-induced cell damage. The biological activities underlying opioid production strongly indicated that opioid production systems are reciprocally dysregulated in PD models (Stefano et al., 2012). Opioids, a class of compounds including morphine and pentazocine, may have neuroprotective effects and act as a promising therapy for neurodegeneration disease.

Because opioids have many protective effect both *in vivo* and *in vitro* PD models and Wnt/β-catenin signaling is involved in the development of PD, we designed the present study to analyze the protective effects of pentazocine in MPP^+^-induced SN4741 cell damage and the importance of Wnt/β-catenin signaling in the pathology of PD.

## Materials and Methods

### Reagents

Fetal bovine serum (FBS), 3-(4,5-dimethylthiazol-2yl)-2,5-diphenyltetrazolium bromide (MTT), Pentazocine, Naloxone and MPP^+^ were purchased from Sigma–Aldrich (St. Louis, MO, USA). Dulbecco’s modified Eagle’s medium (DMEM) was purchased from Gibco (Gaithersburg, MD, USA). TUNEL kit was purchased from Roche (Germany). Recombinant Human Dkk1 was purchased from R&D Systems (Minneapolis, MN, USA). Antibodies to β-catenin, PARP, GAPDH and TH were from Abcam (Cambridge, UK). Antibodies to GSK-3β, p-GSK-3β (ser9), cleaved caspase-3, cleaved caspase-8 and β-actin were from Cell Signaling (Beverly, MA, USA). Anti-mouse-HRP IgGanti-rabbit -HRP IgG were purchased from Pierce Biotechnology (Rockford, IL, USA).

### Cell Culture

SN4741 cells, a mouse embryonic substantial nigra-derived neural cell line, were grown in DMEM containing 1% penicillin, 10% heat-inactivated FBS, 1% glucose and 2 mM glutamine in a humid 5% CO_2_ environment at 33°C (Son et al., 1999). The experiments were performed when cells reached 60%–70% confluence. SN4741 cells were exposed to MPP^+^ at various concentrations (0–120 μM) for 24 h. The cells were also pretreated with Pentazocine (0–50 μM) for 24 h before adding MPP^+^. Dkk1 and Naloxone was added prior to Pentazocine.

### MTT Assay

The cell viability was determined by conventional MTT assay. The SN4741 cells grown in the 96-well microplate were treated with MTT labeling reagents and incubated for 2 h in a humidified incubator at 37°C. Absorbance was determined at 490 nm on a microplate reader (Bio-Rad, Hercules, CA, USA) and cell viabilities were calculated. Results were expressed as percentage of the values of the control cells (control = 100%).

### Western Blot Analysis

Nuclear fractions of protein were extracted according to protocol of Nucleus and Cytoplasmic Protein Extraction Kit (Borong biology, shanghai, china). Western blot was performed with protein lysates obtained from SN4741 cells. The cells in MPP^+^ group were treated with 90 μM MPP^+^ for 24 h. Meanwhile, cells in control group were treated with equal volume of PBS. Cells in MPP^+^+pentazocine group were pretreated with 20 μM pentazocine for 24 h before adding MPP^+^. Moreover, cells in MPP^+^+pentazocine+naloxone or MPP^+^+pentazocine+Dkk1 group were treated with 10 μM naloxone for 15 min or 100 ng/μl Dkk1 for 2 h before adding pentazocine and MPP^+^. Then the protein was separated by SDS-PAGE and transferred to PVDF membranes. The membranes were blocked with 5% BSA in TBST at room temperature for 2 h and incubated with Antibodies to β-catenin (90 kDa), PARP (116 kDa), GAPDH (36 kDa) and TH (56 kDa; Abcam, Cambridge, UK) and Antibodies to GSK-3β (46 kDa), p-GSK-3β (ser9; 46 kDa), cleaved caspase-3 (19 kDa), cleaved caspase-8 (43 kDa) and β-actin (42 kDa; Cell Signaling, Beverly, MA, USA) at 4°C overnight. After three washes in TBST, the membranes were incubated with HRP-conjugated secondary antibodies. Then the membranes were washed with TBST for three times. At last, the protein bands were visualized using chemiluminescence detection.

### Quantitative Real-time PCR

The total RNA was extracted using TRIZOL reagent (Code No. 15596018, Invitrogen), and mRNA was reversely transcribed into cDNA (Code No. RR036A, TAKARA). The reagents used in quantitative real-time PCR were purchased from Applied Biosystems™ (Code No. 4472908, Life Technologies). The products were amplified by a quantitative real-time PCR machine. The expression of target gene was normalized to β-actin mRNA level. The primer sequences were shown in Table 1.

**Table 1:** The primers for real time PCR.

Factors	Primers
TH	Forward 5′-CCTGGAGTACTTTGTGCGCT-3′
	Reverse 5′-GGGAACCAGGGAACCTTGTC-3′
GSK-3β	Forward 5′-TAGTCGAGCCAAGCAGACAC-3′
	Reverse 5′-GACCAGCTGCTTTGCACTTC-3′
β-catenin	Forward 5′- GTCAGTGCAGGAGGCCGA-3′
	Reverse 5′- CTCCATCAGGTCAGCTTGAGT-3′
β-actin	Forward 5′-TATAAAACCCGGCGGCGCA-3′
	Reverse 5′-TCATCCATGGCGAACTGGTG-3′

### Immunocytochemistry

SN4741 cells were fixed with 4% paraformaldehyde solution for 10 min. The cells were then blocked with PBS containing 5% bovine serum albumin (BSA) for 2 h and incubated with 0.1% Triton-100 for 30 min. After three washes in PBS, the cells were incubated with β-catenin primary antibody at 4°C overnight. The next day, the cells were labeled with FITC-conjugated anti-rabbit secondary antibodies for 2 h at room temperature. Then we washed the cells with PBS for three times. The nuclei were labeled with DAPI for 10 min at room temperature. Finally, the cells were washed with PBS for three times and imaged with a Nikon fluorescence microscope.

### TUNEL Staining

The apoptosis was determined using the one-step TUNEL Apoptosis Kit (Beyotime, JS, China). After each designated treatment, SN4741 cells were fixed, permeabilized, and incubated with TUNEL reaction mixture at 37°C for 1 h according to the manufacturer’s protocol. The cells were then stained with DAPI and observed with a laser scanning confocal microscope (C2 Si; Nikon, Japan) after three times washes with PBS.

### Flow Cytometric Analysis

The Annexin V-FITC/PI cell apoptosis detection kit (KeyGen Biotech, Nanjing, China) was used to analyzed the cell apoptosis. After washing with PBS, the SN4741 cells were resuspended in 500 μL of binding buffer and then incubated in 5 μL of Annexin V-FITC for at least 10 min. At last, SN4741 cells were washed with PBS and stained with 10 μL of propidium iodide for 20 min at 37°C in the dark. Data acquisition and analysis were performed in a Becton Dickinson FACSCalibur flow cytometer using the Cell Quest software (USA).

### Transient Transfection and Reporter Assays

SN4741 cells were transfected with TOPFlash/FOPFlash plasmids (200 ng; Millipore, USA) using Lipofectamine 2000 (Invitrogen) in DMEM without FBS. After incubation for 6 h, the primary medium was exchanged for complete DMEM. Cells were exposed to designated treatment 24 h after transfection. One-hundred microliter of lysis buffer was added to the cells which had received designated treatment. Then the cells were transferred to a microcentrifuge tube and incubated at 37°C for 15 min. After 5 s of centrifugation, the supernatant was transferred to a new microcentrifuge which contained luciferase reagent buffer. Luciferase activity was assayed with a dual-luciferase reporter system (Promega).

### Statistical Analysis

All the data were analyzed using one-way analysis of variance (ANOVA) followed by Bonferroni test. All values are expressed as the mean ± SEM. *P* < 0.05 was considered as statistically significant.

## Results

### Effect of Pentazocine on the MPP^+^-Induced Neurotoxicity in SN4741 Cells

**Figure 1:**
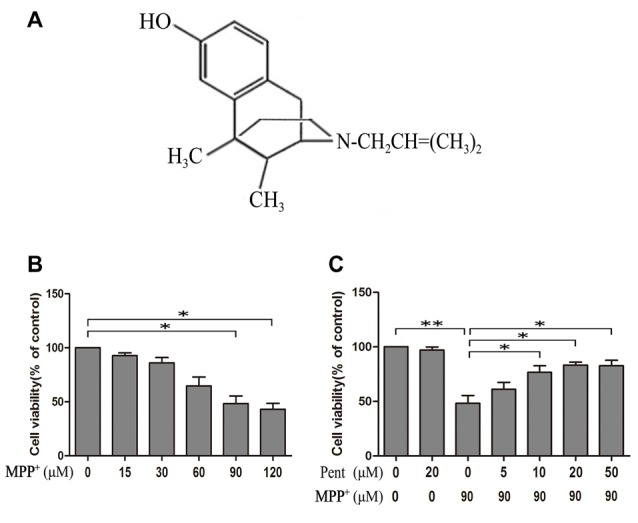
3-(4,5-dimethylthiazol-2yl)-2,5-diphenyltetrazolium bromide (MTT) assay showed the protective effect of pentazocine against 1-methyl-4-phenylpyridinium (MPP^+^)-induced decrease in cell viability in SN4741 cells. **(A)** The chemical structure of pentazocine. **(B)** Effects of MPP^+^ on the cell viability for 24 h at various concentrations (0–120 μM). After different treatments, the SN4741 cells grown in the 96-well microplate were added with MTT labeling reagents and incubated for 2 h in a humidified incubator at 37°C. Absorbance was determined at 570 nm on a microplate reader. **(C)** The effect of pentazocine on MPP^+^-induced cytotoxicity. SN4741 cells were exposed to MPP^+^(90 μM) for 24 h. The cells were also pretreated with Pentazocine (0–50 μM) for 24 h before adding MPP^+^. Then the MTT assay was conducted in different groups (the means ± SEM; *n* = 3; *p < 0.05; **p < 0.01).

Pentazocine is an agonist of the opioid receptor (Figure 1A). To evaluate the effect of MPP^+^ on the cell viability, we treated SN4741 cells with various concentrations of MPP^+^ for 24 h and examine the cell viability using MTT. The results indicated that the viability of cells decreased significantly after treated with 90 μM MPP^+^ for 24 h (Figure 1B). Therefore, exposure of 90 μM MPP^+^ for 24 h was used in subsequent experiments. Then we treated the SN4741 cells with 20 μM pentazocine alone for 24 h to investigate whether pentazocine was solely responsible for the change of cell viability. The results showed no difference on cell viability compared to control group. However, the viability of SN4741 cells was increased when the cells were pretreated with different concentrations (10, 20 and 50 μM) of pentazocine for 24 h before MPP^+^ treatment (Figure 1C). All these results indicated that pentazocine could protect cells from MPP^+^-induced cell viability decrease in a dose-dependent manner.

### Pentazocine Rescued β-Catenin from MPP^+^-Induced Death in SN4741 Cells

**Figure 2:**
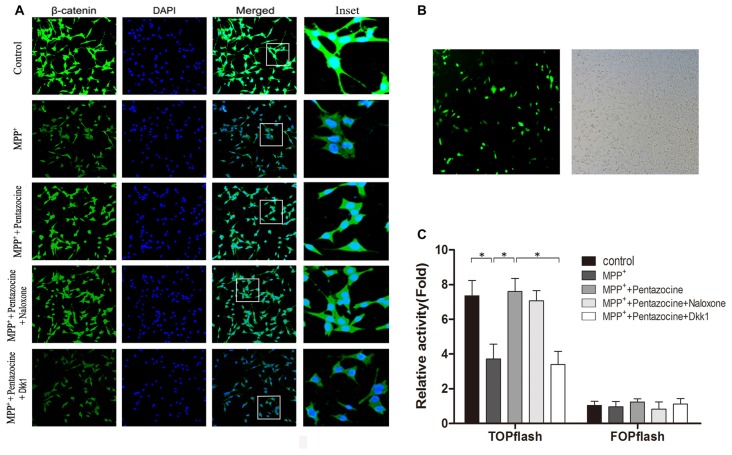
The protective effect of pentazocine on MPP^+^-induced downregulation of Wnt/β-catenin signaling can be ameliorated by Dickkopf-1 (Dkk1) pretreatment. **(A)** The immunofluorescence analysis of β-catenin in SN4741 cells. Cells were pretreated with pentazocine (20 μM) for 24 h before 24 h of MPP^+^ (90 μM) treatment. Moreover, we treated the SN4741 cells with 10 μM naloxone for 15 min or 100 ng/μl Dkk1 for 2 h before adding pentazocine and MPP^+^. **(B)** Transfection efficiency of TOPFlash/FOPFlash plasmids in SN4741 cells was assayed by GFP plasmid. **(C)** SN4741 cells were transfected with TOPFlash/FOPFlash luciferase reporter constructs and were treated as described in **(A)**. Original relative activity means the ratio of luciferase activity in each group compared with the resulting from FOP in MPP^+^ group. Then the original relative activity/cell viability in each group can be regarded as relative activity. (The means ± SEM; *n* = 5; *p < 0.05).

β-catenin protein plays an important role in the canonical Wnt signaling, and many evidences indicate that change of the canonical Wnt signaling is involved in the neuronal degeneration of PD. To investigate whether pentazocine affects the expression of β-catenin protein, the cells were pretreated with pentazocine (20 μM) for 24 h before MPP^+^ treatment. Moreover, we treated the SN4741 cells with 10 μM naloxone for 15 min or 100 ng/μl Dkk1 for 2 h before adding pentazocine and MPP^+^. The β-catenin protein level in each group was measured by immunocytochemistry assay (Figure 2A). The results suggested that MPP^+^ induced a down-regulation in the protein level of β-catenin. However, the β-catenin levels increased when cells were pretreated with pentazocine compared with cells in MPP^+^ group. The adding of naloxone or Dkk1 demonstrated that only Dkk1 was associated with the abrogation of protective effect of pentazocine, whereas naloxone was not. To examine whether the disturbance of β-catenin leads to the change of TCF/LEF related gene expression, we used a TOPFlash/FOPFlash reporter assay. The transfection efficiency of plasmid in SN4741 cells was determined by GFP plasmid (Figure 2B). The TOPFlash/FOPFlash plasmids were transfected into SN4741 cells. Then cells were divided into five groups. MPP^+^ group was treated with 90 μM MPP^+^ for 24 h. Meanwhile, cells from control group were treated with equal volume of PBS. Cells from MPP^+^+pentazocine group were pretreated with 20 μM pentazocine for 24 h before adding MPP^+^. Moreover, cells from MPP^+^+pentazocine+naloxone or MPP^+^+pentazocine+Dkk1 group were treated with 10 μM naloxone for 15 min or 100 ng/μl Dkk1 for 2 h before adding pentazocine and MPP^+^. The results showed that luciferase activity was significantly decreased in the SN4741 cells administrated with MPP^+^. We also observed that pretreatment of pentazocine before adding MPP^+^ significantly promoted the luciferase activity compared with cells in MPP^+^ group. The adding of naloxone or Dkk1 before pentazocine and MPP^+^ treatment also indicated that only Dkk1 was related to the abrogation of protective effect of pentazocine, whereas naloxone was not (Figure 2C).

### The Level of P-GSK-3β (ser9) and Nuclear β-Catenin Changed after Adding MPP^+^

**Figure 3:**
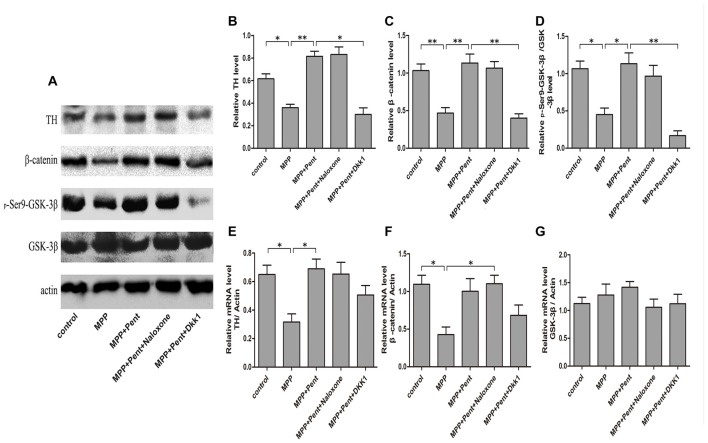
Pentazocine effectively rescued β-catenin from MPP^+^-induced decline via canonical Wnt pathway. **(A)** Cells were treated as described in Figure 2A. Western blots was performed for the TH, β-catenin, GSK-3β, and _p_-GSK-3β (ser9) measurements. **(B–D)** Relative amounts of TH/actin, β-catenin/actin, _p_-GSK-3β (ser9)/GSK-3β were quantified (the means ± SEM; *n* = 3; *p < 0.05; **p < 0.01). **(E–G)** RNA was obtained from the SN4741 cells and analyzed with quantitative real-time PCR. The values of TH, β-catenin and GSK-3β were normalized to that of β-actin (the means ± SEM; *n* = 3; *p < 0.05).

**Figure 4:**
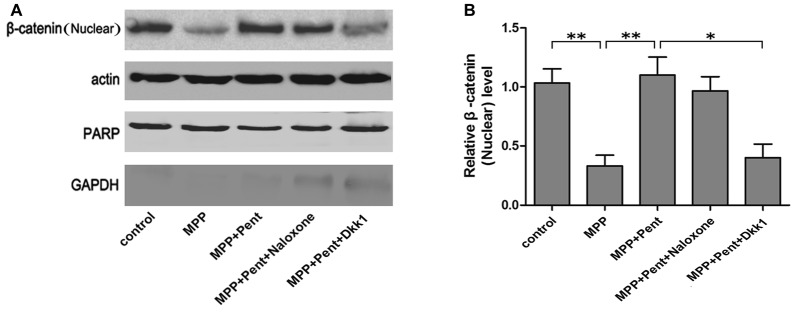
The protective effects of pentazocine on MPP^+^-induced decline of β-catenin could be abrogated by Dkk1 in SN4741 cell nucleus. **(A)** Western blots was performed for the β-catenin in nucleus. **(B)** Quantification graphs indicating the ratio of β-catenin (nucleus) to actin (the means ± SEM; *n* = 3; *p < 0.05; **p < 0.01).

To test whether the downstream molecules of Wnt/β-catenin signaling waschanged, we used same treatment paradigm. Then we measured the TH, β-catenin, GSK-3β, p-GSK-3β (ser9) and β-catenin levels by Western blot (Figures 3A–D) and quantitative real-time PCR (Figures 3E–G). The specificity of primers were verified in NCBI website (Supplementary Figure S1). The results showed that inhibition of the canonical Wnt/β-catenin signaling was found in MPP^+^ treated group, for the p-GSK-3β (ser9) and β-catenin were down-regulated. Pentazocine can reverse the down-regulation of canonical Wnt/β-catenin signaling and protect the SN4741 cells from MPP^+^ injury, which verified that the down-regulation of canonical Wnt/β-catenin signaling was involved in the MPP^+^-induced neurotoxicity in SN4741 cells. The use of Dkk1 abrogated the effect of pentazocine in the up-regulation of Wnt/β-catenin signaling. However, naloxone showed no influence on Wnt/β-catenin signaling in the SN4741 cells treated with pentazocine and MPP^+^. We also tested the expression of β-catenin in nucleus by Western blot and the data were consistent with the change of β-catenin in total cells. PARP was detected in the nuclear fractions of protein, whereas GAPDH was not. This result indicated that the nuclear fractions were isolated successfully (Figures 4A,B).

### Pentazocine Attenuated MPP^+^-Induced Cells Apoptosis via Wnt Signaling

**Figure 5:**
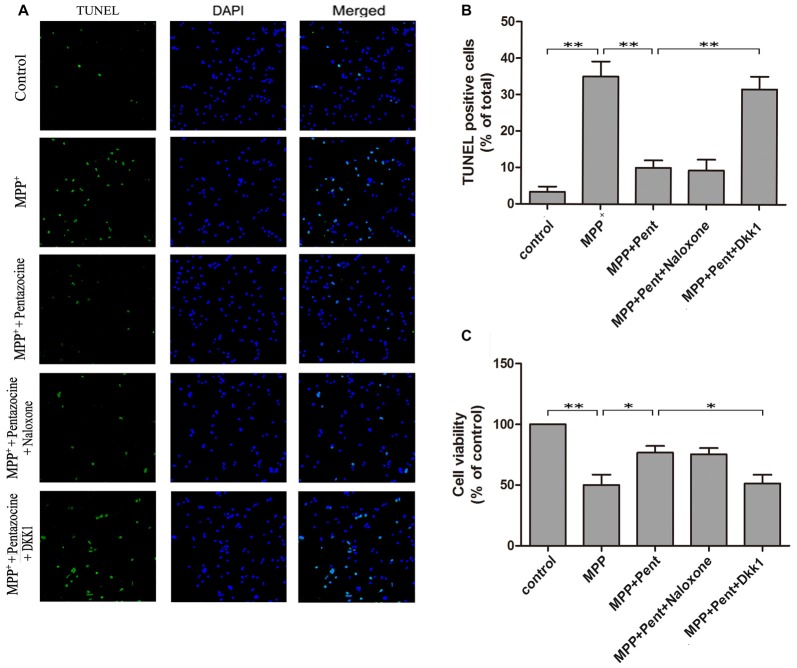
Pentazocine attenuated the MPP^+^-induced SN4741 cells apoptosis via Wnt/β-catenin signaling. Cells were treated as described in Figure 2A. **(A)** The SN4741 cells apoptosis was analyzed by the TUNEL assay. Cells were also stained with DAPI as counterstain for the cell nuclei. **(B)** Quantification of the percentage of apoptotic SN4741 cells in different groups (the means ± SEM; *n* = 3; **p < 0.01). **(C)** SN4741 Cell viability in different groups was measured by MTT assay (the means ± SEM; *n* = 3; *p < 0.05; **p < 0.01).

TUNEL staining and Western blots were used to evaluate the effect of pentazocine on MPP^+^-induced SN4741 cells apoptosis. The results indicated that the number of TUNEL-positive SN4741 cells increased after MPP^+^ treatment (Figures 5A,B). However, fewer TUNEL-positive SN4741 cells were observed when cells were pretreated with pentazocine for 24 h before adding MPP^+^ compared with the cells treatment with MPP^+^ alone. The protective effect of pentazocine was abrogated when the SN4741 cells were treated with 100 ng/μl Dkk1 for 2 h before adding pentazocine and MPP^+^. Moreover, naloxone showed no influence on the protective effect of pentazocine in the SN4741 cells pretreated with pentazocine and MPP^+^. This finding was further supported by the MTT assay in different groups (Figure 5C).

**Figure 6:**
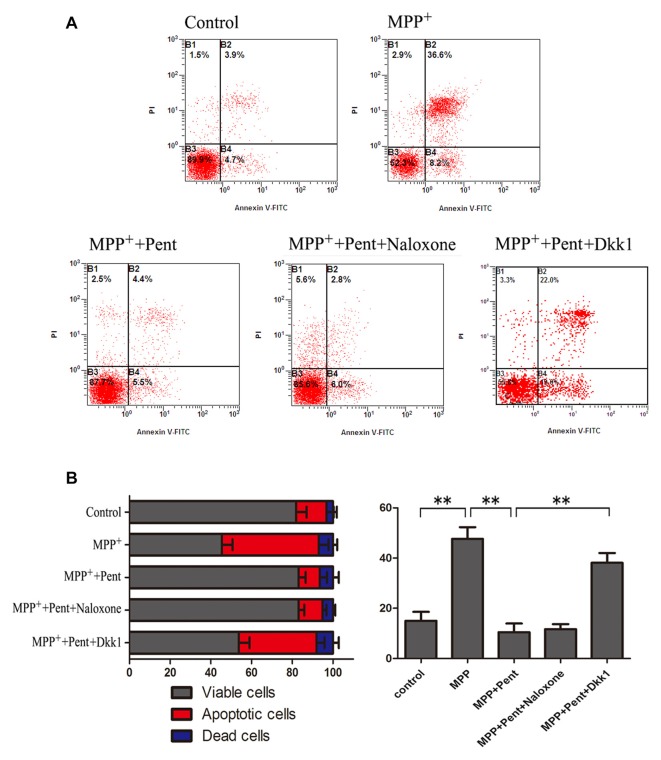
Dkk1 could abrogate the protective effect of pentazocine on MPP^+^-induced SN4741 cells apoptosis. Cells were treated as described in Figure 2A. **(A)** Annexin V-FITC/PI was performed to examine the cell apoptosis. **(B)** Percentages of apoptotic SN4741 cells in every group (the means ± SEM; *n* = 3; **p < 0.01).

**Figure 7:**
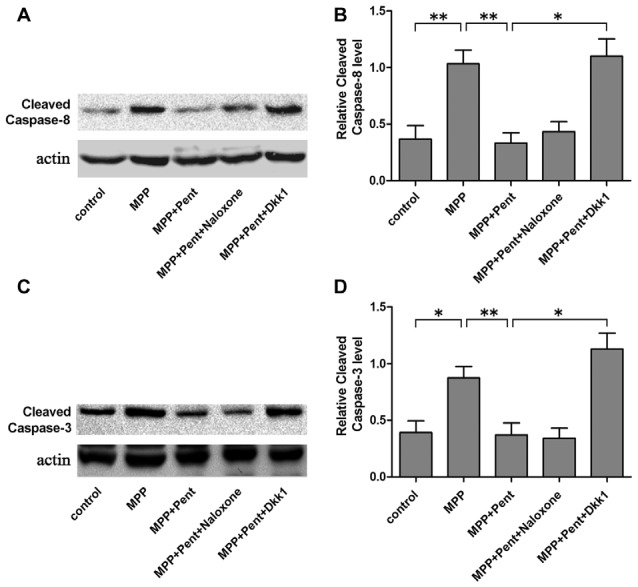
Expression of cleaved caspase-8 and cleaved caspase-3 in different groups. **(A)** The level of cleaved caspase-8 in SN4741 cells was analyzed by Western blots. Cells were treated as described in Figure 2A. **(B)** The ratio of cleaved caspase-8 to actin was determined by quantitative analysis (the means ± SEM; *n* = 3; *p < 0.05; **p < 0.01). **(C)** Western blots showed the level of cleaved caspase-3 in the different group of SN4741 cells. **(D)** The ratio of cleaved caspase-3 to actin was determined by quantitative analysis (the means ± SEM; *n* = 3; *p < 0.05; **p < 0.01).

To strengthen the evidence, SN4741 cells were stained with the Annexin V-FITC/PI. After exposure to MPP^+^ for 24 h, the apoptotic cells were increased compared to that of the control group. Pretreatment of pentazocine for 24 h remarkably abolished this effect. Besides, the protective effect of pentazocine was abrogated when the SN4741 cells were treated with 100 ng/μl Dkk1 for 2 h (Figures 6A,B). The Western blot showed that pentazocine exposure reversed the MPP^+^ induced increase of cleaved caspase-8 and cleaved caspase-3 level in SN4741 cells. To test whether pentazocine exerted protections through Wnt/β-catenin signaling, cells were pretreated with Dkk1 to block the activity of Wnt/β-catenin signaling. Then we found that the protectiveeffect of pentazocine against MPP^+^-induced increase of cleaved caspase-8 and cleaved caspase-3 level was abrogated in cells incubated with 100 ng/μl Dkk1 for 2 h. However, the effect of pentazocine against MPP^+^-induced increase of cleaved caspase-8 and cleaved caspase-3 level could not be abrogated in cells pretreated with 10 μM naloxone for 15 min (Figures 7A–D).

## Discussion

PD is a common progressive neurodegenerative disease with loss of dopaminergic neurons in substantia nigra and is clinically characterized by gait dysfunction, tremors, bradykinesia and rigidity (Takagi and Takahashi, 2005; Pickrell et al., 2011). Currently, only the symptomatic therapies of PD are efficacious. Most therapies cannot stop or retard the apoptosis of dopaminergic neurons in substantia nigra (Toulouse and Sullivan, 2008). Therefore, drugs that can slow or halt the neurodegeneration in PD patients are expected to have a tremendous impact (Fukui et al., 2010; Fahn and Poewe, 2015). Naloxone is an antagonist that has been treated as an antidote to opioids through mu-opioid receptor. Morphine, an opioid isolated from the plant extracts of the opium poppy, has been showed to protect SH-SY5Y human neuroblastoma cells against 6-OHDA-induced cell damage (Elyasi et al., 2014). However, whether pentazocine, another agonist of opioid receptor with low risk of addiction, provides neuroprotection against the MPP^+^-induced cell damage was unknown prior to this research.

MPP^+^, which serves as a selective toxin for dopaminergic neurons, has been studied in experimental models of PD (Friedemann et al., 2016). It has been reported that MPP^+^ stimulates superoxide formation and induces oxidative damage to proteins (Blum et al., 2001). The MTT assay showed that the adding of MPP^+^ in SN4741 cells resulted in dose dependent viability decrease. Furthermore, the number of TUNEL-positive cells was increased after MPP^+^ treatment. In contrast, the pretreatment of pentazocine before adding MPP^+^ resulted in an increase of cell viability and decrease of TUNEL-positive cells compared with that in response to the MPP^+^ alone. These data suggests that pentazocine alleviates MPP^+^-induced SN4741 cell apoptosis.

The canonical Wnt/β-catenin signaling is negatively regulated by a range of molecules, which act either extracellularly by affecting the interaction between Wnt ligands and its receptor complex, or intracellularly by interfering with signal transduction (Chen et al., 2009; Paluszczak et al., 2015; Wo et al., 2016). Dkk1 has been regarded as one of the extracellular antagonist of canonical Wnt/β-catenin signaling (Zhou et al., 2016). It has been reported that Wnt/β-catenin signaling is important for the pathogenesis of many neurodegenerative diseases, including ischemic insults (Cappuccio et al., 2005), patients of AD (Caricasole et al., 2004), and temporal lobe epilepsy (Busceti et al., 2007). Dun et al. (2012) have reported that down-regulation of the canonical Wnt pathway contributes to neurodegeneration in the 6-OHDA-lesioned rats. Here, we conducted this study in the models of PD and an obvious decrease of β-catenin was observed in SN4741 cells treated with MPP^+^.

Previous studies have indicated that the Wnt/β-catenin signaling pathway is activated after opioids administration in the rat spinal cord and cerebral cortex (Novikova et al., 2005; Dunbar et al., 2006). Furthermore, morphine has been showed to induce the secretion of Wnt signaling proteins (Jaremko et al., 2014). Besides, the opioids have many beneficial roles against the damage in central nervous system (Cui et al., 2011). In the present study, Western blots and Immunocytochemistry showed that pentazocine could reverse the decline of β-catenin induced by MPP^+^ in SN4741 cells. The adding of naloxone or Dkk1 before pentazocine treatment demonstrated that the down-regulation of β-catenin induced by MPP^+^ could not be rescued by pentazocine when the Wnt receptors were blocked. Therefore, we supposed that only the Wnt receptors were related to the rescue of β-catenin expression after pentazocine treatment, whereas opioid receptor was not. More importantly, the Western blots, MTT assay, and TUNEL staining showed that pentazocine exposure reversed the MPP^+^ induced apoptosis of SN4741 cells. Indeed, the protective effect of pentazocine was abrogated when the SN4741 cells were treated with Dkk1 before adding pentazocine and MPP^+^, indicating that pentazocine functions in a Wnt/β-catenin signaling dependent manner (Tan et al., 2015).

In conclusion, the data presented here shows that pentazocine reverses the apoptosis induced by MPP^+^ in SN4741 cells. Moreover, the protective effects of pentazocine are associated with an up-regulation of the β-catenin levels. These results suggest that pentazocine may be a potential therapy of the neurodegeneration in PD via up-regulation of the canonical Wnt/β-catenin signaling. Besides, these findings will be useful to explain the molecular mechanisms of PD. Pentazocine should next be examined in animal models before being regarded as a candidate for clinical trial.

## Author Contributions

JW and JG participated in the design of the study. HW and GZ performed the statistical analysis. DF and YL carried out immunofluorescent study. WG and KT performed PCR. GG and LG helped draft the manuscript. All authors read and approved the final manuscript.

## Conflict of Interest Statement

The authors declare that the research was conducted in the absence of any commercial or financial relationships that could be construed as a potential conflict of interest.
