# Supplementary figures and images for: Pentazocine Protects SN4741 Cells Against MPP+-Induced Cell Damage via Up-Regulation of the Canonical Wnt/β-Catenin Signaling Pathway
Source: Front Aging Neurosci. 2017 Jun 14;9:196. doi: 10.3389/fnagi.2017.00196 (PMC5469889; doi:10.3389/fnagi.2017.00196)

**Supplementary Figure1.** The specificity of primers in NCBI website.


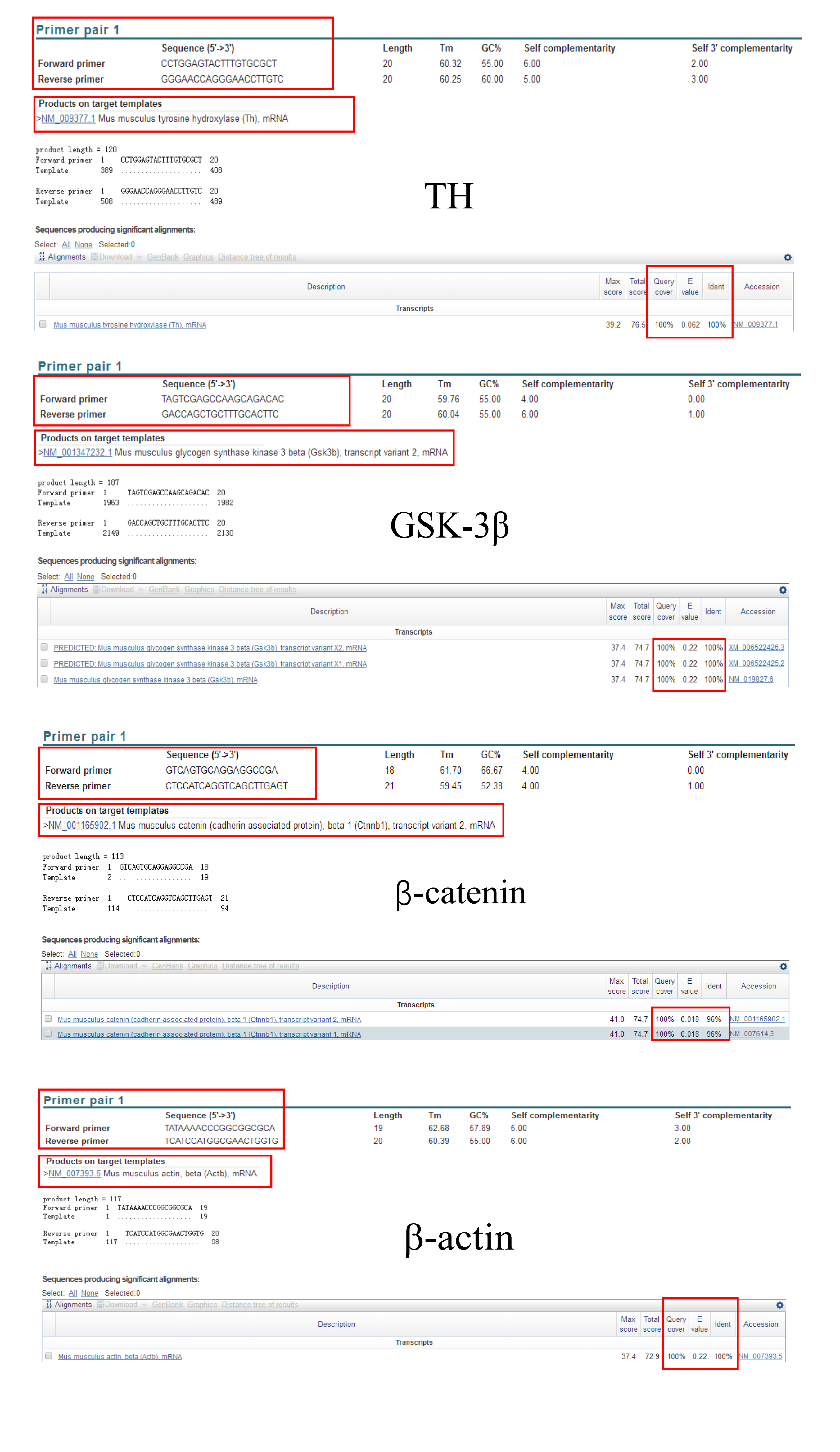

Supplement: Supplementary file 1 [file Data_Sheet_1.doc]
